# The Status of Assessments and Treatments for Osteoporosis in Patients 5 Years after Total Hip Arthroplasty: A Cross-Sectional Survey of 194 Post-THA Patients

**DOI:** 10.1155/2019/1865219

**Published:** 2019-03-31

**Authors:** Keiji Kamo, Hiroaki Kijima, Koichiro Okuyama, Shin Yamada, Natsuo Konishi, Hitoshi Kubota, Hiroshi Tazawa, Naohisa Miyakoshi, Yoichi Shimada

**Affiliations:** ^1^Akita Rosai Hospital, Japan; ^2^Akita Hip Research Group, Japan; ^3^Department of Orthopedic Surgery, Akita University Graduate School of Medicine, Japan

## Abstract

**Background:**

Assessments for osteoporosis in patients who have undergone total hip arthroplasty (THA) are very important with respect to the clinical results. However, few studies have investigated the status of the assessments and treatments for osteoporosis in post-THA patients. The purpose of this multicenter study was to investigate the status of assessments and treatments for osteoporosis in post-THA patients.

**Methods:**

The results of a self-report questionnaire and the medical records of 194 post-THA patients over 40 years of age who visited the outpatient departments of the five hospitals participating in the study were analyzed.

**Results:**

A total of 125 patients (64.4%) had been examined for osteoporosis, and 69 patients (35.6%) had never been assessed for osteoporosis. It was assumed, based on the questionnaire results, that 50 (40%) of the 125 patients should have been receiving treatment for osteoporosis. Forty-five (90%) of these 50 patients were actually taking medication for osteoporosis at the time of the investigation. Overall, a total of 58 (29.9%) patients were receiving treatment for osteoporosis.

**Conclusions:**

The present survey revealed that 64.4% of post-THA patients had been evaluated for osteoporosis. Moreover, while 40% of post-THA patients over 40 years of age may require treatment for osteoporosis, only 29.9% were actually receiving treatment.

## 1. Introduction

The proportion of elderly people is increasing globally. Of all the advanced countries, Japan is at the center of this aging society phenomenon. Yoshimura et al. have estimated that nearly 970,000 people (160,000 men, 810,000 women) ranging in age from 40 to 79 years develop osteoporosis in Japan annually [1]. While some racial differences are likely to be present, similar incidences of osteoporosis can be expected in other countries. The prevalence of degenerative hip disorders or traumatic disorders requiring surgical intervention is higher in the elderly population, and it is assumed that the number of older patients who choose to undergo THA to improve their quality of life will increase [2, 3]. Thus, orthopedic surgeons will likely use THA as a surgical intervention to treat these disorders much more frequently in the aging societies of the future.

Osteoporosis is one of the main causes of intraoperative periprosthetic femoral fracture and reduced initial stability of the implant in cementless THA [4, 5]. Osteoporosis also increases the chances of a periprosthetic fracture after THA with uncemented stem [5, 6]. A periprosthetic femoral fracture negatively affects not only the results of THA but also a mortality rate [6–8]. Thillemann et al. reported that the 10-year cumulative implant revision rate in primary THA patients with osteoporosis from the Danish Hip Arthroplasty Registry, in which the implant fixation technique of THA included cemented, uncemented, and hybrid fixation, was 8.3% [9], which is significantly higher than the rate in patients without osteoporosis. Therefore, the assessments and treatments for osteoporosis in patients who undergo THA, especially with uncemented stem, are crucial to improve or maintain the clinical results. While several studies have demonstrated the importance of the degree of osteoporosis in patients who undergo THA, very little attention has been paid to the pre- and postoperative status of the assessments and treatments for osteoporosis. To the best of our knowledge, few reports have investigated osteoporosis in post-THA patients. The purpose of the present multicenter study involving five affiliated hospitals in Akita Prefecture, Japan, was to evaluate the status of assessments and treatments for osteoporosis in post-THA patients.

## 2. Materials and Methods

This cross-sectional study of the assessments and treatments for osteoporosis in post-THA patients was approved by the Ethics Committee of the authors' affiliated institutions. A total of 194 post-THA patients (246 hips) over 40 years of age who visited the outpatient department of the five participating hospitals between April and May 2016 were investigated. The status of assessments and treatments for osteoporosis were ascertained using a self-report questionnaire (Table 1) and the patients' medical records. All 194 patients were enrolled in this survey.

## 3. Results

The mean age of the patients was 70 years (range, 44–92 years), and there were 26 men and 168 women. The mean duration after THA was 62 months (1–408 months). Reasons for undergoing THA included osteoarthritis (163 cases), rheumatoid arthritis (8 cases), osteonecrosis of the femoral head (8 cases), hip fracture (7 cases), and rapidly destructive coxarthropathy (4 cases). The original diseases of the remaining four cases were not clearly recorded. In terms of the type of implant fixation technique, there were 183 uncemented, 5 cemented, and 6 hybrid fixation of THA in this survey.

Of the 194 patients who were enrolled in this survey, 125 (64.4%) patients reported that they had been evaluated for osteoporosis in the questionnaire. It was ascertained that 40.0% (50/125) of the patients assessed for osteoporosis required treatment, while 55.2% (69/125) did not. The remaining six patients answered “unknown” regarding their osteoporotic assessment. Ninety percent (45/50) of the patients that required osteoporotic treatment were taking medication at the time of the present survey. One patient (1/50) had never been treated for osteoporosis. Of the patients assessed for osteoporosis, 8.0% (4/50) had stopped receiving treatment before the time of the present investigation. Of the patients that were not evaluated for osteoporosis, 8.7% (6/69) were being treated for osteoporosis at the time of the present survey. In total, 58 (29.9%) of the 194 patients were being treated for osteoporosis at the time of the present survey. Bisphosphonate, vitamin D, selective estrogen receptor modulator, teriparatide, calcium, and denosumab were the medications being taken for treatment of osteoporosis in 33, 18, 9, 6, 5, and 4 cases, respectively. Of the 163 OA patients, 108 (66.3%) had been assessed for osteoporosis. Thirty-nine (36.1%) of the 108 patients assessed for osteoporosis reported that they were required treatment in the result of the assessment. Forty-six (28.2%) of the 163 OA patients were being treated for osteoporosis at the time of the present investigation. Of the remaining 31 patients who received THA for the other reasons, 17 (54.8%) had been assessed for osteoporosis. Eleven (64.7%) of the 17 patients assessed for osteoporosis reported that they were required treatment in the result of the assessment. Twelve (38.7%) of 31 THA patients for the other reasons were being treated for osteoporosis at the time of the present investigation.

## 4. Discussion

In the present survey, the status of assessments and treatments for osteoporosis in post-THA patients was investigated in five affiliated hospitals in Akita Prefecture, Japan. Results show that approximately two-thirds of the patients had been examined for osteoporosis, and that more than 40.0% of the patients required treatment for osteoporosis. However, only 29.9% (58/194) of the patients were actually undergoing treatment for osteoporosis. Previous studies have reported an incidence of osteoporosis of 25–28% in women with hip arthritis scheduled for or undergoing THA [10, 11]. The present survey included patients that were followed up for an average of 5 years after THA. To our knowledge, although previous studies have described preoperative status, no other study has described the postoperative assessments of osteoporosis or osteoporosis treatment. The patients in the present study were older and the prevalence of osteoporosis was higher than in past reports [10]. It is speculated that, given the length of the follow-up period (mean, 62 months; range, 1–408 months), some patients without osteoporosis at the time of THA may have developed osteoporotic conditions by the time of the survey. Consequently, it is crucial to examine the status of osteoporosis not only at the time of THA surgery, but also throughout the period of post-THA follow-up.

In previous studies related to THA and osteoporosis medication, Yamasaki et al. reported that risedronate reduced periprosthetic bone resorption after cementless THA [12]. Iwamoto et al. reported that alendronate monotherapy and combined therapy using alendronate and alfacalcidol both prevent periprosthetic bone mineral density loss after THA [13]. In addition, a few reports have demonstrated that bisphosphonate use is associated with a lower risk for revision surgery in patients with osteoporosis undergoing primary THA [13, 14]. Therefore, treatment for osteoporosis in patients that have undergone THA is very important for improving the long-term results of THA.

In the present study, while approximately 40% of patients were considered to need osteoporotic treatment, only 30% were being treated for osteoporosis at the time of the investigation. This discrepancy must not be overlooked. It is assumed that some patients (about 10%) that were being followed up as outpatients after THA were neither diagnosed with nor being treated for osteoporosis despite presenting with osteoporotic conditions.

Approximately 90% of the patients diagnosed with conditions requiring treatment for osteoporosis were actually receiving treatment for osteoporosis. This indicates that adherence to osteoporosis therapies is extremely good when patients who have undergone THA are informed that they have osteoporosis and require treatment. Six patients (8.7%) among the patients who had not been evaluated for osteoporosis were receiving treatment for osteoporosis. It is speculated that patients receiving long-term steroids are treating osteoporosis for the prevention of osteoporosis. Gleeson et al. reported that periodic follow-up interaction between patients and health care professionals appeared to be beneficial for improving adherence and persistence with osteoporosis medications [15]. Patients that have had THA are required to visit the hospital regularly for radiographs of their hips. It is speculated that this periodic follow-up of patients after THA improves their adherence to osteoporosis therapies.

Patients who underwent or are scheduled for THA will inevitably age, so it is essential that these patients receive the appropriate osteoporotic treatment at the appropriate time. Regarding the adherence of patients to osteoporosis therapies after THA, there is a possibility that the pre- and postoperative assessments of osteoporosis indirectly improve the long-term results of THA.

The present study had several limitations. First, this investigation only used a questionnaire and medical records. Second, the method used for osteoporotic assessment was unknown. Osteoporosis should be diagnosed based on the presence of a fragility fracture and measurements of bone mineral density [16]. However, the results of this research clarified the current situation regarding the evaluation of and treatment for osteoporosis in patients who underwent THA within the previous 5 years. Future studies should investigate whether treatment for osteoporosis prevents periprosthetic fractures and improves the long-term results of THA.

In conclusion, more than 40% of patients over the age of 40 years who underwent THA may require treatment for osteoporosis. However, less than 30% of patients were being treated for osteoporosis, as only about two-thirds of patients were actually evaluated for osteoporosis. Because the adherence to osteoporosis therapies in post-THA patients is very good, thorough evaluation of osteoporosis after THA may lead to better long-term results of THA.

## Figures and Tables

**Table 1:** Questionnaire about assessments and treatments for osteoporosis given to post-THA patients.

(1) Have you ever been evaluated for osteoporosis?	(a) Yes (b) No

(2) If you have been evaluated for osteoporosis, what was the result?	(a) Treatment required (b) No treatment required (c) Unknown

(3) Have you ever been treated for osteoporosis?	(a) Yes (b) No (c) I was previously being treated, but I am not being treated now.

(4) If you are being treated for osteoporosis, what kind of treatment are you receiving?	

## Data Availability

The data used to support the findings of this study are included within the article.
